# Study on the Potential Biomarkers of Maternal Urine Metabolomics for Fetus with Congenital Heart Diseases Based on Modified Gas Chromatograph-Mass Spectrometer

**DOI:** 10.1155/2019/1905416

**Published:** 2019-05-06

**Authors:** Donghua Xie, Yingchun Luo, Xiyue Xiong, Mingxing Lou, Zhiyu Liu, Aihua Wang, Lili Xiong, Fanjuan Kong, Yichao Wang, Hua Wang

**Affiliations:** ^1^Department of Information Management, Maternal and Child Health Hospital of Hunan Province, 58 Xiangchun Road, Changsha, Hunan 410078, China; ^2^Department of Ultrasonography, Maternal and Child Health Hospital of Hunan Province, 58 Xiangchun Road, Changsha, Hunan 410078, China; ^3^Department of Pediatric Rehabilitation, Maternal and Child Health Hospital of Hunan Province, 58 Xiangchun Road, Changsha, Hunan 410078, China; ^4^NHC Key Laboratory of Birth Defect for Research and Prevention (Hunan Provincial Maternal and Child Health Care Hospital), Hunan 410008, China

## Abstract

**Background:**

There has been significant research on the genetic and environmental factors of congenital heart defects (CHDs), but few causes of teratogenicity, especially teratogenic mechanisms, can be clearly identified. Metabolomics has a potential advantage in researching the relationship between external factors and CHD.

**Objective:**

To find and identify the urinary potential biomarkers of pregnancy (including in the second and third trimesters) for fetuses with CHD based on modified gas chromatograph-mass spectrometer (GC-MS), which could reveal the possibility of high-risk factors for CHD and lay the foundation for early intervention, treatment, and prevention.

**Methods:**

Using a case-control design, we measured the urinary potential biomarkers of maternal urine metabolomics based on GC-MS in a population-based sample of women whose infants were diagnosed with CHD (70 case subjects) or were healthy (70 control subjects). SIMCA-P 13.0 software, principal component analysis (PCA), orthogonal partial least squares-discriminant analysis (OPLS-DA), Wilcoxon-Mann-Whitney test, and logistics regression were used to find significant potential biomarkers.

**Result:**

The 3D score graph of the OPLS-DA showed that the CHD and control groups were fully separated. The fitting parameters were R^2^x=0.78 and R^2^y=0.69, and the forecast rate was Q^2^=0.61, indicating a high forecast ability. According to the ranking of VIPs from the OPLS-DA models, we found 34 potential metabolic markers with a VIP > 1, and after two pairwise rank sum tests, we found 20 significant potential biomarkers, which were further used in multifactor logistic regressions. Significant substances, including 4-hydroxybenzeneacetic acid (*OR*=4.74, 95% CI: 1.06-21.06), 5-trimethylsilyloxy-n-valeric acid (*OR*=15.78, 95% CI: 2.33-106.67), propanedioic acid (*OR*=5.37, 95% CI: 1.87-15.45), hydracrylic acid (*OR*=6.23, 95% CI: 1.07-36.21), and uric acid (*OR*=5.23, 95% CI: 1.23-22.32), were associated with CHD.

**Conclusion:**

The major potential biomarkers in maternal urine associated with CHD were 4-hydroxybenzeneacetic acid, 5-trimethylsilyloxy-n-valeric acid, propanedioic acid, hydracrylic acid, and uric acid, respectively. These results indicated that the short chain fatty acids (SCFAs) and aromatic amino acid metabolism may be relevant with CHD.

## 1. Introduction

Congenital heart defects (CHDs) are the most common types of birth defects (BDs), accounting for nearly one-third of all BDs [1, 2]. Only 15% of heart defects can be attributed to a known cause [3]. The causes of CHD are complicated and include environmental factors, genetic susceptibilities, and their interactions [4]. However, given the complicated causes of CHDs, there is little research on how multiple external influencing factors could induce CHDs in fetuses or how the pathogenesis of CHD correlates with external factors. Metabolomics is a new tool that could enlarge the effects of external environmental factors [5] and, thereby, indirectly deduce the mechanisms of how external environmental factors induce CHDs. Compared to genomics, which requires the disclosure of personal information and is expensive, metabolomics is lower cost and noninvasive [6]. Metabolomics have been used in the field of obstetrics and gynecology which may allow physicians to identify possible associated etiologies that affect the mother during pregnancy and lead to associated complications affecting the offspring [5, 7]. Therefore, metabolomics research is an important approach for studying some mechanisms of CHD and is a future developmental trend [8].

Given the nature of the sample, the purpose of the experiment, and the chemical and physical properties of the metabolites, different analysis and identification techniques were selected.

Presently, the most commonly used separation methods include nuclear magnetic resonance (NMR), liquid chromatography-mass spectrometry (LC-MS), gas chromatography-mass spectrometry (GC-MS), etc. [9, 10]. GC-MS is a mass spectrometry technology used for the separation and identification of volatile substances [11]. It is complemented by a relatively complete National Institute of Standards and Technology (NIST) database and high throughput, sensitivity, selectivity, and resolution capabilities [12]. Relative to LC-MS, GC-MS has certain advantages in the identification of metabolites [13]. The difficulties and key of GC-MS are sample pretreatment and processing. We self-developed metabolomics methods for sample pretreatment (patent number: 2012101142462) on the basis of traditional GC-MS. This method optimizes the impurity removal and chemical derivatization process, improves the derivatization efficiency, and protects components that are not stable and easily volatilize at high temperatures. The GC-MS method can be used to analyze amino acids, organic acids, fatty acids, amides, nucleosides, sugars, and other small molecules at the same time, including in particular, semivolatile metabolites of SCFAs, such as hydracrylic acid, propanedioic acid, and so forth [14].

To our knowledge, until now, there has been only one study that included a comprehensive metabolomic analysis for the prediction of fetuses with CHD in the first trimester [15]. It is worth mentioning that it was reported that the maternal gut microbiota in late pregnancy and early lactation was stable [16]. However, it is difficult to find CHD cases in the first trimester using four-dimensional echocardiography which is the only diagnostic method available, excluding some very complicated cases. In the findings, we present the search for potential metabolic biomarkers during midterm pregnancy (including the second and third trimesters) based on advanced GC-MS and discuss the association between CHDs and the mother's metabolism in vivo.

## 2. Materials and Methods

### 2.1. Patient Recruitment and Sample Collection

This study was approved by the Ethics Committee of Maternal and Child Care, Hospital of Hunan Province. Each recruited pregnant woman signed a written consent.

The pregnant women included in the study had no history of antibiotic use in the last three months and no history of abortion, diabetes, gestational diabetes, digestive system diseases, liver and renal disease, thyroid disease, chromosomal disorders, family history, or picky eating. The research period was the optimal time for screening for CHD, during gestational weeks 22-24 and 30-32. The patients were diagnosed with a CHD fetus by highly professional doctors with four-dimensional echocardiography. The controls were diagnosed as normal, and health was confirmed after delivery at the Maternal and Child Care Hospital of Hunan Province. Urine samples were collected in clean containers from both the last urination of the night and the first urination of the morning, and the exact time of collection was recorded. The urine from the night needed to be frozen in the refrigerator. The next morning, the first urination was sent to the test center within two hours, along with the urine from the previous night. The two urine samples from the same individual were combined into one sample. Each urine sample was enriched with filter paper and stored at -80°C immediately after collection until analysis.

### 2.2. Sample Pretreatment

The samples were pretreated as described in the research of Xiong X Y [17]. Urine samples were briefly thawed at room temperature and centrifuged (at 3000 g) for 10 min, and 100 *μ*L urine samples (containing 2.5 mmol/L creatinine) were first treated with 30.0 *μ*L urease (1.2 U/*μ*L) at 37°C for 30 min to remove interfering urea and then spiked with heptadecanoic acid (0.5 mg/mL, 50 *μ*L). Proteins, including the added urease, were precipitated with 800 *μ*L ethanol and removed after 15 min of centrifugation (12000 r/min). Forty microliters of 0.04 mol/L hydroxylamine hydrochloride and 60 *μ*L of 0.05 mol/L Ba(OH)_2_ were added to the deproteinized solution, and the mixture was then incubated at room temperature for 15-20 min. Subsequently, the mixture solution was evaporated to dryness, and the compounds in the dried residue were converted into TMS derivatives with 100 *μ*L of MSTFA/TMCS (100:1) and analyzed by GC-MS (ZL 201210114246.2). Quality control (QC) samples, used for monitoring the repeatability and stability of the analytical method, were prepared by mixing all the urine samples and tested 5 times repeatedly.

### 2.3. GC-MS Analysis

An Agilent GC-MS system (7890-5975C) was used to analyze the derivative samples. A sample (1 *μ*L) was injected with a split ratio of 20:1 into the GC and then separated with a fused silica HP-5 capillary column (60 m, 0.25 mm inside diameter, 0.25 *μ*m thickness of the inner liquid in the column). The injector temperature was set to 250°C. High purity nitrogen was used as a carrier gas at a constant flow rate of 1.5mL/min. The column temperature was initially kept at 60°C for 2 min, ramped up to 300°C at 5.5°C/min, and then held for 10 min. The interphase and ion source temperatures were 280°C and 230°C, respectively. Ions were generated by electronic impact (EI) at 70 eV. Masses were acquired from 50 to 1000* m*/*z*. The drift of the retention time of each peak was minimized by locking heptadecanoic acid at 36.00 min using retention time locking technology (RTL, Agilent). GC-MS ChemStation software was used for auto-acquisition of GC total ion chromatograms (TICs) and fragmentation patterns. Each compound had a fragmentation pattern composed of a series of split molecular ions; the mass charge ratios and their abundance were compared with a standard mass chromatogram in the NIST (National Institute of Standards and Technology) mass spectra library by the ChemStation software. Peaks with a similarity index greater than 70% were assigned compound names, and compounds with a matching rate < 70% were excluded.

### 2.4. Statistical Analysis

After GC-MS analysis, each sample was represented by a GC-MS TIC, and the ion peak areas of compounds were integrated. The results were expressed as ratios to the urinary creatinine concentration. Statistical analysis was used for the comparison of the metabolite levels to determine significant differences between the CHD and control groups.

Data were exported from the GC-MS ChemStation software, including all information regarding the TICs after the above process, into SIMCA-P 13.0. PCA and OPLS-DA were used to differentiate the samples. PCA scores are unsupervised as a basis for OPLS-DA which is supervised. R^2^ and Q^2^ provide a measure of model reliability of PCA and OPLS-DA [18]. The OPLS-DA model was assessed by the intercepts of R^2^ and Q^2^ in Permutation test to avoid the overfitting. The criteria for model validity include two conditions: (1) all Q^2^ values on the permuted data set to the left are lower than the Q^2^ values on the actual data set to the right and (2) the regression line (line joining the point of observed Q^2^ to the centroid of a cluster of permuted Q^2^ values) has a negative value of intercept on the* y*-axis [19]. Through the variable importance for the projection (VIP) of the OPLS-DA model, the potential biomarker was found with VIP >1. A larger VIP indicated a more important biomarker. The Wilcoxon-Mann-Whitney test was used to verify the significant difference in potential biomarkers between the CHD and control groups. Based on the Wilcoxon-Mann-Whitney test, logistic was used to screen the biomarkers with *α*_in_=0.05 and *α*_out_=0.1. The two-class criteria of all independent variables in the logistic model were confirmed through the ROC curve.* P* values of <0.05 were considered to be statistically significant.

## 3. Results

### 3.1. Study Population

A total of 70 patients (CHDs were diagnosed by 4D color ultrasound) treated at the Ultrasonic Department, Hunan Provincial Maternal and Child Health Care Hospital between 2016.7.1 and 2017.7.30 were included. An additional 70 qualifying normal pregnant women were randomly selected. There was no statistical significance in the maternal age, gestational weeks, progestation BMI (kg/m^2^), BMI (kg/m^2^) at inspection time, and increase in BMI (kg/m^2^) (*P*>0.05) (Table 1).

Among the 70 patients, there were 27 VSDs (38.57%), 10 TOFs (13.73%), 8 aortic abnormalities (11.43%), and so on (Table 2).

### 3.2. Screening Potential Urinary Biomarkers

**Figure 1:**
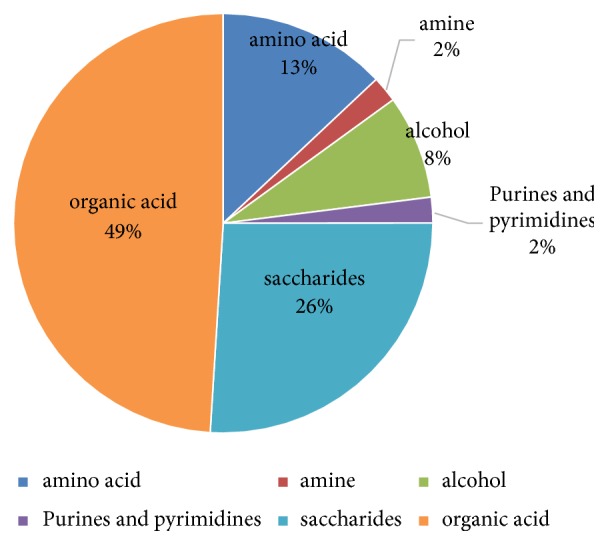
The constituent ration of 130 compounds.

Before analysis, some data treatments were made. Firstly, after NIST library searching and credibility screening, there were 220 metabolites. Secondly, delete the metabolites which are not present in the 80% of the population. Finally, 130 metabolites were retained which mainly were organic acid (49%), saccharides (26%), amino acid (13%), and so on (Figure 1). Thirdly, these metabolites were parametric but not normal. After some transformations including log transformation, these data still not to be normal.

**Figure 2:**
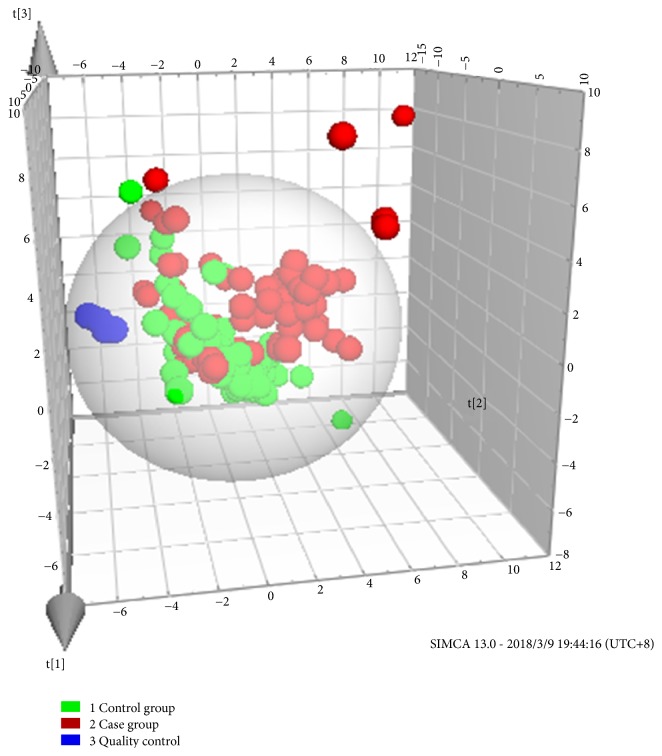
The PCA-3D score graph of maternal urine metabolism profile between the case group and control group.

The separation between groups (Figure 2) can be observed on the PCA 3D score, and the model fitting parameter was R^2^x=0.57 with a forecast rate of Q^2^=0.46. Although some samples were overlapped in the PCA score plot, samples showed separation pattern between groups, indicating that metabolite profiles of two groups were different.

**Figure 3:**
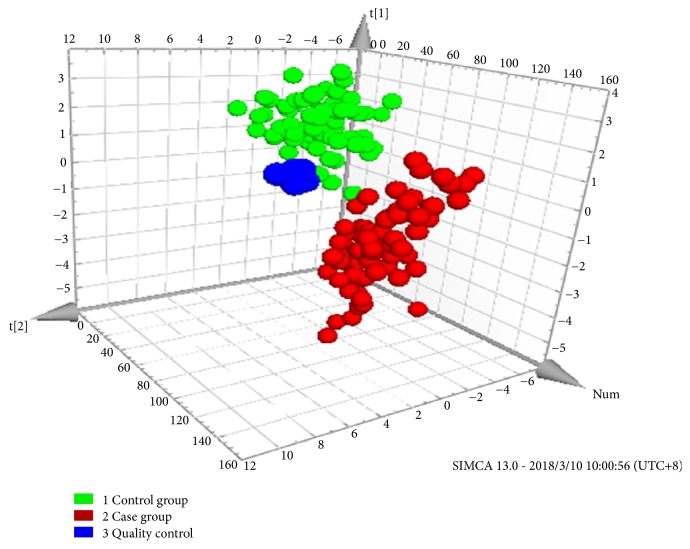
The OPLS-DA-3D score graph of maternal urine metabolism profile between the case group and control group.

**Figure 4:**
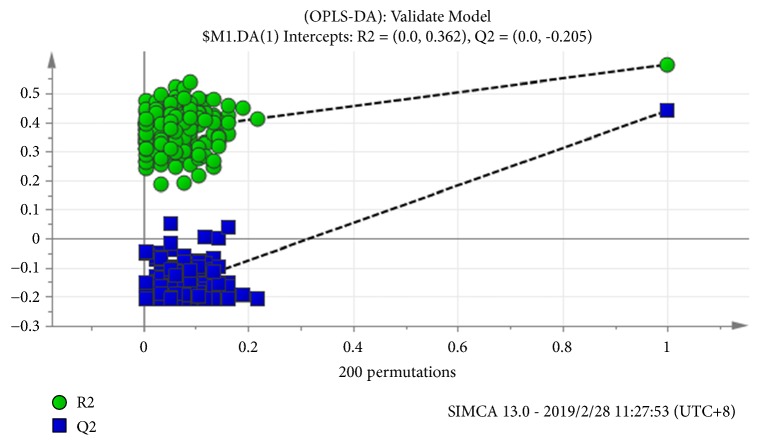
Permutation test of OPLS-DA.

**Figure 5:**
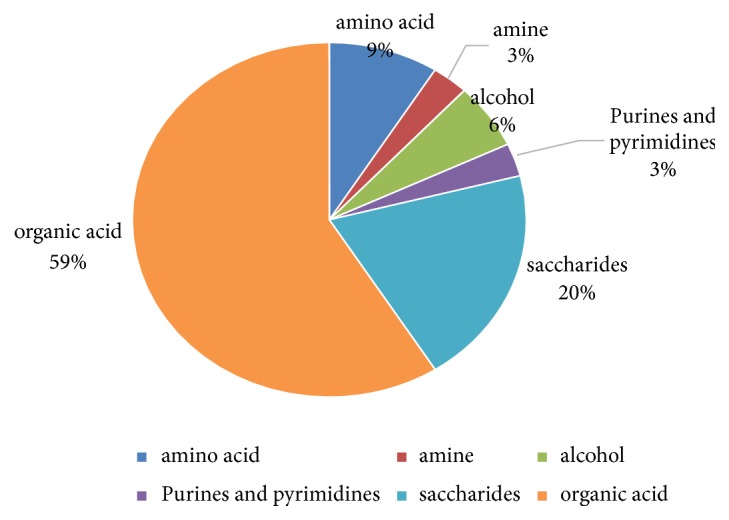
The constituent ration of 34 compounds.

OPLS-DA modeling yielded two principal components, and 3D score maps (Figure 3) showed the CHD and control groups were fully separated. Through a permutation test repeated 200 times, Q^2^ and R^2^ values were found to be higher than their original values, proving suitability and validity of this model (Figure 4). The fitting parameters were R^2^x=0.78 and R^2^y=0.69, and the forecast rate was Q^2^=0.61, indicating a high forecast ability. According to the ranking of VIPs from the OPLS-DA models, we found 34 potential metabolic biomarkers with VIP >1 (Table 3), which mainly were organic acid (59%), saccharides (20%), amino acid (9%), and so on (Figure 5).

After the Wilcoxon-Mann-Whitney test, we found 20 significant potential biomarkers, which are listed in Table 3 (*P*<0.05). We also listed their diagnostic predictive value through the ROC curve, which is the two-class standard. If the value of the potential biomarker was more than the predictive value, it was judged to be “1.” If it was less than the predictive value, it was judged to be “0” in the logistic model. The results of the logistic regression showed that the significant substances, including 4-hydroxybenzeneacetic acid (*OR*=4.74, 95% CI: 1.06-21.06), 5-trimethylsilyloxy-n-valeric acid (*OR*=15.78, 95% CI: 2.33-106.67), propanedioic acid (*OR*=5.37, 95% CI: 1.87-15.45), hydracrylic acid (*OR*=6.23, 95% CI: 1.07-36.21), and uric acid (*OR*=5.23, 95% CI: 1.23-22.32), were associated with CHD (Table 4). The five compounds were further qualitatively analyzed by comparing their retention time and fragment-ion of the chromatograms between the urine sample and the corresponding standards (Figures6–11; see Supplementary Material). All of the data support the identification of the five compounds as 4-hydroxybenzeneacetic acid, 5-trimethylsilyloxy-n-valeric acid, propanedioic acid, hydracrylic acid, and uric acid, respectively.

## 4. Discussion

Our study showed that the separation between groups could be observed using PCA and OPLS-DA modeling. We found 34 potential metabolic markers with a VIP >1, and after two pairwise rank sum tests, we found 20 significant potential biomarkers. The significant substances were 4-hydroxybenzeneacetic acid, 5-trimethylsilyloxy-n-valeric acid, hydracrylic acid, propanedioic acid, and uric acid after further multifactor logistic regression. This result is similar to the research of Bahado-Singh RO, which concluded that abnormal lipid metabolism was a significant feature of CHD pregnancy [15]. The main meaning and the relative mechanism of these potential makers as follows.

In our study, 4-hydroxybenzeneacetic acid was higher in the CHD group than in the control group (*OR*=4.74,95% CI: 1.06-21.06). As reported, tyrosine is converted from phenylalanine under the action of intestinal bacteria [20], and tyrosine decarboxylation is further converted into 4-hydroxybenzeneacetic acid [21]. It can be seen that the increase in 4-hydroxybenzeneacetic acid in the case group may be due to a disorder of microecological bacteria in the pregnant mother of the CHD fetus or the increasing of tyrosine.

5-Trimethylsilyloxy-n-valeric acid from enterogenous SCFAs was higher in the CHD group than in the control group (*OR*=15.78, 95% CI: 2.33-106.67). SCFAs mainly include acetic acid, propionic acid, butyric acid, pentanoic acid, and so on [22]. After traveling to the liver through the bloodstream, SCFAs participate in biotransformation reactions, such as oxidation, reduction, hydrolysis, and conjugation [23]. Thus, all kinds of SCFAs produce a large amount of intermediate or terminal metabolites, such as propionic acid, which produces acrylic acid, beta-hydroxypropionic acid, malonic acid, propionylglycine, etc. [23, 24]. Valeric acid produces 5-trimethylsilyloxy-n-valeric acid, valerylglycine, and so on [23]. Without special dietary conditions, 5-trimethylsilyloxy-n-valeric acid may be derived primarily from valeric acid via omega-oxidative metabolism (valeric acid is similar to the omega-oxidative metabolic pathway of valproate (VPA) [25]. The excessive concentration of 5-trimethylsilyloxy-n-valeric acid in the urine of the case group may indicate valproate metabolic disorders in the pregnant mother of the CHD fetus. When there are too many SCFAs, such as valeric acid, CHD can be induced by exerting physiological effects similar to VPA, such as deacetylase inhibitors [26, 27], secondary demethylation enhancement [28], and folic acid antagonists [29].

Hydracrylic acid and propionic acid levels were higher in the CHD group than in the control group (*OR*=6.23, 95% CI: 1.07-36.21), (*OR*=5.37, 95% CI: 1.87-15.45). Hydracrylic acid and malonic acid are mainly derived from intestinal-derived propionic acid [30]. Propionic acid enters the epithelial cells of the intestine to provide energy, dehydrogenation into acrylic acid, addition of water to produce Hydracrylic acid, and then dehydrogenation, thiolysis reactions into malonate [31]. In the study, the increase in hydracrylic acid and propionic acid in the maternal urine of mothers with CHD fetuses may be caused by too much propionic acid in the intestinal microecology. Propionic acid, one of the most important components of SCFAs, is the major product of the fermentation of undigested carbohydrates, such as oligosaccharides, nonstarch polysaccharides, and resistant starches by Clostridium species IV and XIV [32]. When there are too much propionic acid, CHD can be induced by exerting physiological effects similar to VPA. In additional, this suggests that the CHD group may be associated with an excess of Clostridium genera IV and XIV in the thick-walled bacteria in the intestinal tract.

Uric acid was higher in the CHD case group than in the normal control group (*OR*=5.23, 95% CI: 1.23-22.32). Uric acid is the final product of purine metabolism. Dysfunction of purine metabolism, abnormal energy metabolism, and renal excretion of uric acid can cause either an increase (hyperuricemia) or decrease (hypouricemia) in plasma uric acid concentration [33]. More studies have shown that metabolic diseases, such as obesity, can affect the risk of hyperuricemia. Changes in intestinal flora may affect purine metabolism by altering certain body characteristics [33]. Animal experiments have also shown that probiotics can reduce the levels of serum uric acid in mice [34], mainly due to the reduction of serum endotoxin and the increase of xanthine oxidase activity caused by probiotic treatment, which in turn reduces uric acid production and decreases serum uric acid levels [35]. It can be speculated that the occurrence of CHD may be related to a disorder in purine metabolism caused by the imbalance of intestinal flora in the mother (the specific mechanism is not yet clear).

In our study, the pretreatment technology of GC-MS is simpler and produces a more accurate result, especially in semivolatile metabolites from SCFAs, such as hydracrylic acid, propanedioic acid, etc. In addition, several statistical methods were combined to seek out potential biomarkers in the maternal urine. This result could make up for the lack of ultrasounds. The estimated current prenatal ultrasound screening in developed countries detects only 30-50% of cases with CHD [36]. Many pregnant women do not receive an ultrasound in time, and the results are affected by pregnancy weeks, doctor's experience, apparatus, and obesity. Of course, our study has some limitations at the same time. First, for the case-control study, we could not make an inference of causal association between the significant biomarkers and CHD but it gave us some clue about the mechanism of CHD. Second, the samples size is limited; we could not make subgroup comparison. We will collect more samples and make subgroup comparison in the next step. Thirdly, although the model of OPLS-DA was validated, there may be some overoptimistic view of the separation between groups. So, the study was a discovery-phase worthy of further study.

## 5. Conclusions

In sum, the major potential biomarkers in maternal urine associated with CHD were 4-hydroxybenzeneacetic acid, 5-trimethylsilyloxy-n-valeric acid, hydracrylic acid, propanedioic acid, and uric acid. The occurrence of CHD was modestly associated with the preternatural gut microbiota of pregnant mothers and the metabolism of SCFAs. These are new directions for CHD research and more energy for research is needed.

## Figures and Tables

**Table 1:** The comparison of basic features between case group and control group.

	Maternals' age	Gestational weeks	Progestation BMI (kg/m^2^)	BMI (kg/m^2^) at inspection time	Increasing BMI (kg/m^2^)
Control group	30.58 ± 4.17	30.76 ± 2.71	20.76 ± 2.85	24.54 ± 3.39	3.79 ± 2.53
Case group	28.62 ± 5.02	28.27 ± 4.96	20.88 ± 3.94	25.46 ± 5.26	4.57 ± 3.59
*p*	0.644	0.514	0.876	0.392	0.295

**Table 2:** The constitue of case group.

Types of CHD	case	Proportion (%)
VSD (ventricular septal defects)	27	38.57
TOF(Tetralogy of Fallot)	10	13.73
Aortic abnormalities (including stenosis, dividing, right side aortic arch)	8	11.43
Complex CHD	5	7.14
AVSD(atrioventricular septal defect)	5	7.14
Pulmonary arterial abnormality(including stenosis, Atresia and left or right heart dysplasia)	3	4.29
endocardial cushion defect	3	4.29
Tricuspid regurgitation	3	4.29
Single atrium/single ventricle	4	5.71

DORV(Right ventricular double outlet)	2	2.86

total	70	100

**Table 3:** Wilcoxon-Mann-Whitney test of 34 potential biomarkers between case group and control group.

NO	Potential biomarkers	VIP	Control group			Case group			Z	P	diagnostic values
			median	Minimum value	Max value	median	Minimum value	Max value			
1	1,4-butanediol	3.11	0.04	0.00	1.66	0.08	0.01	1.56	0.762	0.448	—
2	tartaric acid	2.56	2.36	0.00	20.00	7.42	0.00	121.68	1.540	0.126	—
3	4-Hydroxybenzeneacetic acid	2.27	4.20	0.00	33.21	18.07	0.00	137.18	5.777	0.000	6.87
4	5-Trimethylsilyloxy-n-valeric acid	2.24	5.91	0.00	12.01	14.51	0.00	137.18	5.698	0.000	0.48
5	citramalic acid	2.02	6.04	0.00	61.29	2.21	0.00	26.21	-3.080	0.003	3.11
6	2-hydroxyvaleric acid	1.95	0.62	0.00	48.31	1.28	0.00	49.50	0.798	0.426	—
7	aconitic acid	1.87	4.67	0.00	40.00	10.00	0.00	70.00	4.615	0.000	35.01
8	2,4-dihydroxybutyric acid	1.83	0.28	0.00	1.10	2.09	0.00	7.33	4.423	0.000	0.22
9	D-galactose	1.79	2.09	0.00	20.00	3.97	0.00	10.00	2.338	0.021	21.01
10	hippuric acid	1.78	10.00	0.00	30.00	10.00	0.00	30.00	1.375	0.171	—
11	1,4-dihydroxybutyric acid	1.62	2.09	0.00	30.00	11.90	0.00	40.00	1.916	0.058	—
12	*α*- ketoglutarate	1.60	30.00	0.00	210.00	30.00	0.00	290.00	1.484	0.140	—
13	Ethanamine	1.56	10.00	0.00	30.00	10.00	0.00	30.00	1.879	0.062	—
14	uracil	1.47	0.89	0.00	4.54	0.00	0.00	12.88	3.525	0.001	3.84
15	hydracrylic acid	1.44	0.00	0.00	15.90	6.71	0.00	56.00	3.401	0.001	0.63
16	L- actamic acid	1.37	0.00	0.00	9.25	0.95	0.00	75.66	2.790	0.006	1.14
17	3-hydroxyphenylacetic acid	1.33	31.90	0.00	435.88	74.71	0.00	771.96	2.446	0.016	0.11
18	3-hydroxy butyric acid	1.26	0.00	0.00	4.20	0.04	0.01	11.99	2.805	0.006	0.01
19	pyruvate	1.25	0.00	0.00	0.00	0.00	0.00	51.17	2.975	0.004	0.28
20	stearate	1.23	0.63	0.00	4.11	0.60	0.00	8.17	0.035	0.972	—
21	malonic acid	1.23	0.00	0.00	16.81	12.59	0.00	320.00	2.704	0.008	0.96
22	2,3-dihydroxypropanoic acid	1.22	0.00	0.00	10.00	0.00	0.00	10.00	2.281	0.024	11.01
23	D-galactitol	1.22	0.00	0.00	20.00	2.09	0.00	40.00	2.377	0.020	15.01
24	oxlate	1.21	0.00	0.00	0.06	0.00	0.00	0.77	1.757	0.083	—
25	D- sorbofuranose	1.14	7.31	0.00	40.00	12.09	0.00	70.00	1.134	0.259	—
26	2-keto-l-gluconic	1.14	0.00	0.00	10.00	10.00	0.00	20.00	2.157	0.033	5.01
27	HPHPA	1.12	31.90	0.00	435.88	74.71	0.00	771.96	2.630	0.010	58.81
28	L- sorbofuranose	1.11	1.09	0.00	10.00	2.87	0.00	10.00	0.724	0.470	—
29	L-glycine	1.07	5.59	0.00	33.16	2.55	0.00	18.75	2.605	0.010	0.65
30	uric acid	1.07	0.55	0.00	5.53	1.02	0.00	12.51	2.199	0.030	0.95
31	L-erythrose	1.05	0.00	0.00	30.87	1.25	0.00	41.63	1.448	0.150	—
32	2,3-Hydroxybutanediol	1.03	15.90	0.00	240.00	45.76	0.00	220.00	2.281	0.024	5.01
33	D-fructose	1.02	0.22	0.00	4.55	0.23	0.00	1.74	1.612	0.110	—
34	L-threonine	1.01	30.00	0.00	210.00	30.00	0.00	290.00	1.879	0.062	—

remarks: 3-(3-hydroxyphenyl)-3-hydroxypropionic acid: HPHPA

**Table 4:** Multifactor logistic regression of 20 potential biomarkers between case group and control group.

Potential biomarkers	Wald X^2^	*P*	*OR*	95%CI	
				Lower limit	Upper limit
4-Hydroxybenzeneacetic acid	4.169	0.041	4.74	1.06	21.06
5-Trimethylsilyloxy-n-valeric acid	8.006	0.005	15.78	2.33	106.67
hydracrylic acid	4.144	0.042	6.23	1.07	36.21
propanedioic acid	9.744	0.002	5.37	1.87	15.45
uric acid	4.990	0.025	5.23	1.23	22.32
Constant	31.312	0.000	0.00		

## Data Availability

If someone needs the data, s/he could contact Donghua Xie by email “2210685350@qq.com”.

## Abbreviations

CHD: Congenital heart defects
GC-MS: Gas chromatograph-mass spectrometer
PCA: Principal component analysis
OPLS-DA: Orthogonal partial least squares-discriminant analysis
SCFAs: The short chain fatty acid
BDs: Birth defects
NMR: Nuclear magnetic resonance
LC-MS: Liquid chromatography-mass spectrometry
GC-MS: Gas chromatography-mass spectrometry
NIST: National Institute of Standards and Technology
QC: Quality control
TICs: Total ion chromatograms
VIP: Important for the projection
VSD: Ventricular septal defects
TOF: Tetralogy of Fallot
DORV: Right ventricular double outlet
AVSD: Atrioventricular septal defect
HPHPA: 3-(3-Hydroxyphenyl)-3-hydroxypropionic acid
VPA: Valproate.

## Supplementary Materials

Supplementary Materials Figure 6: chromatograms of the five compounds in standard solution (black) and urine sample (red). Figure 7: electron-impact mass spectrum of hydracrylic acid TMS derivative. Figure 8: electron-impact mass spectrum of propanedioic acid TMS derivative. Figure 9: electron-impact mass spectrum of 5-trimethylsilyloxy-n-valeric acid TMS derivative. Figure 10: electron-impact mass spectrum of 4-hydroxybenzeneacetic acid TMS derivative. Figure 11: electron-impact mass spectrum of uric acid TMS derivative.
