# Supplementary material for: Study on the Potential Biomarkers of Maternal Urine Metabolomics for Fetus with Congenital Heart Diseases Based on Modified Gas Chromatograph-Mass Spectrometer
Source: Biomed Res Int. 2019 May 6;2019:1905416. doi: 10.1155/2019/1905416 (PMC6526572; doi:10.1155/2019/1905416)
Supplement: Supplementary Materials — Figure 6: chromatograms of the five compounds in standard solution (black) and urine sample (red). Figure 7: electron-impact mass spectrum of hydracrylic acid TMS derivative. Figure 8: electron-impact mass spectrum of propanedioic acid TMS derivative. Figure 9: electron-impact mass spectrum of 5-trimethylsilyloxy-n-valeric acid TMS derivative. Figure 10: electron-impact mass spectrum of 4-hydroxybenzeneacetic acid TMS derivative. Figure 11: electron-impact mass spectrum of uric acid TMS derivative. [file 1905416.f1.pdf]

The identification of the five compounds

Figures 6-11 show the further qualitative analysis of the five metabolites by comparing their retention time and fragment-ion of the chromatograms between the urine sample and the corresponding standards, respectively.

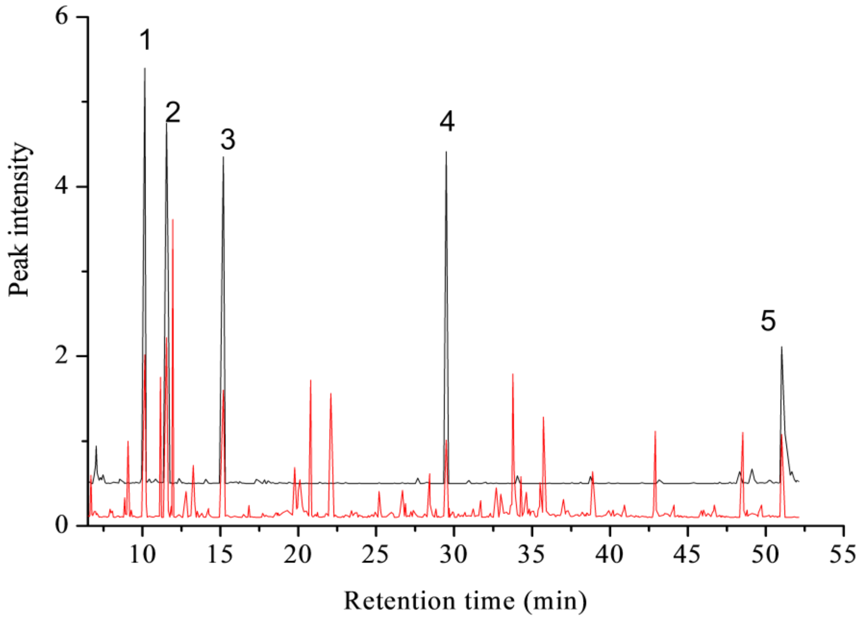

(4). 4-hydroxybenzeneacetic acid, (3). 5-Trimethylsilyloxy-n-valeric acid, (1) hydracrylic acid, (2).propanedioic acid ,(5)uric acid

Fig 6 Chromatograms of the five compounds in standard solution (black) and urine sample (red)

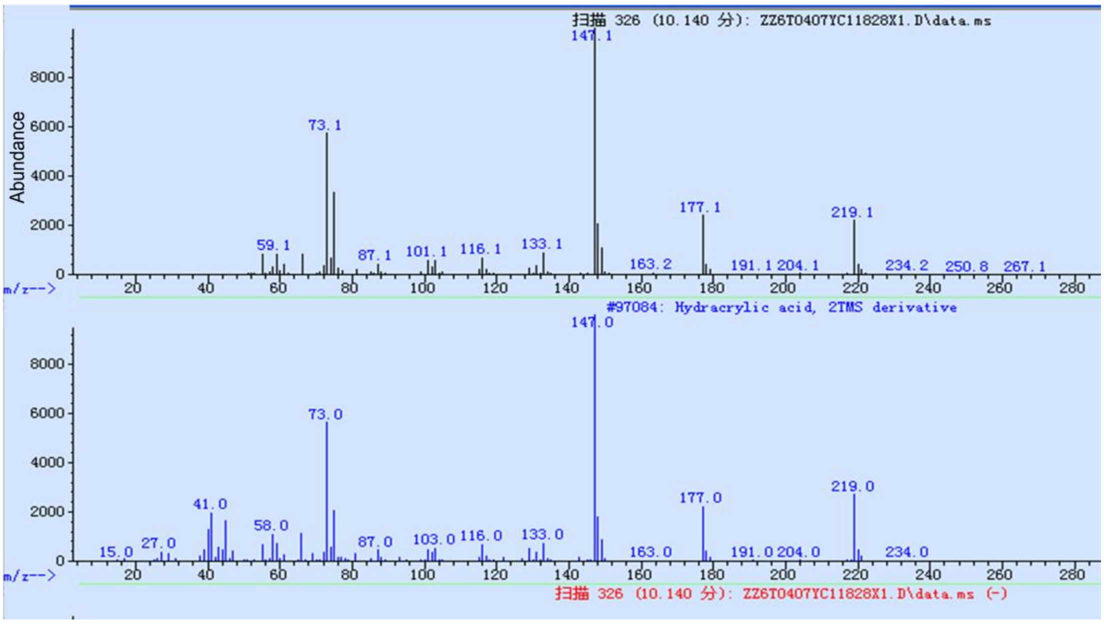

Fig 7 Electron-impact mass spectrum of hydracrylic acid TMS derivative.

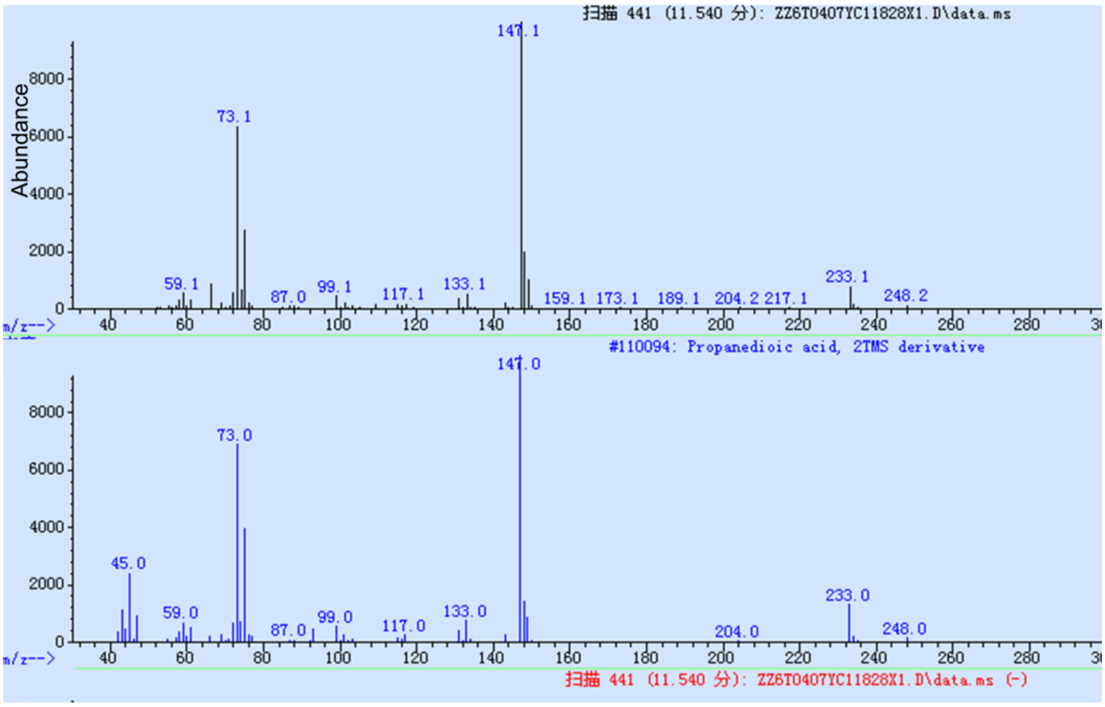

Fig 8 Electron-impact mass spectrum of propanedioic acid TMS derivative.

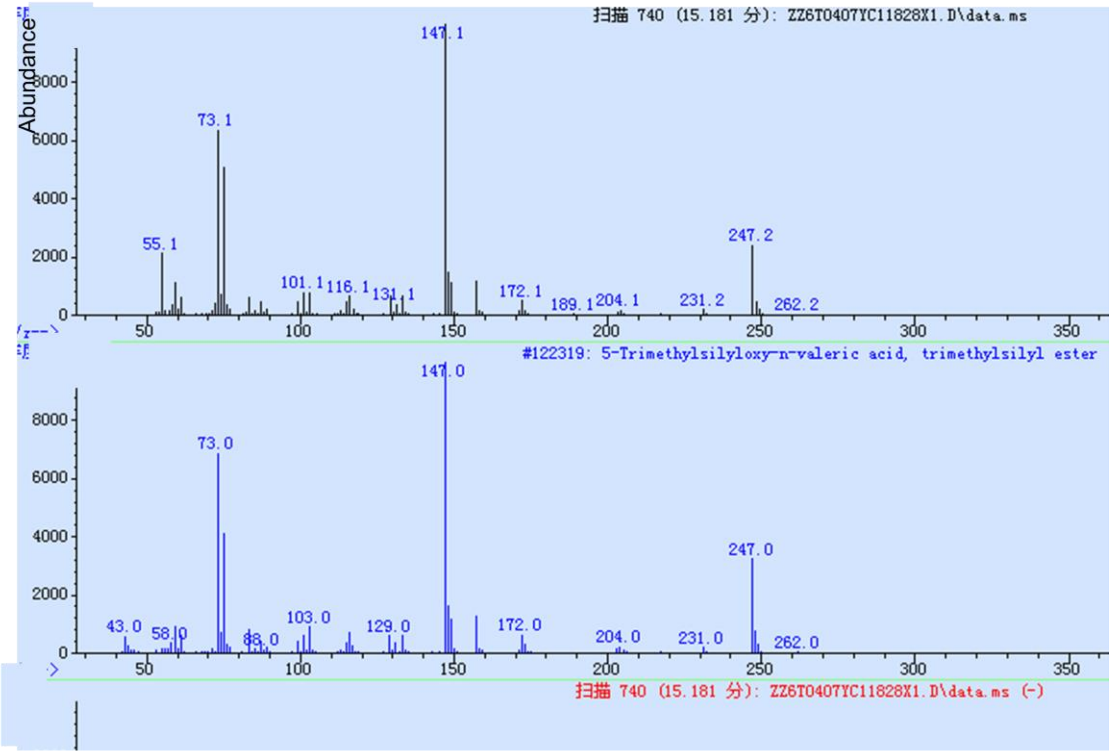

Fig 9 Electron-impact mass spectrum of 5-Trimethylsilyloxy-n-valeric acid TMS derivative.

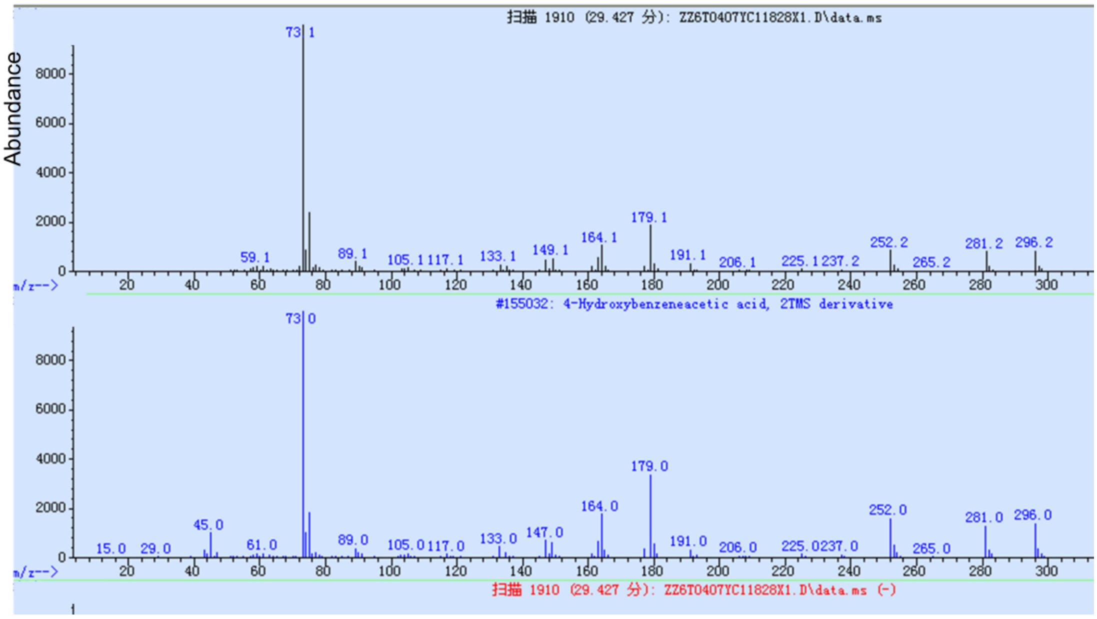

Fig 10 Electron-impact mass spectrum of 4-hydroxybenzeneacetic acid TMS derivative

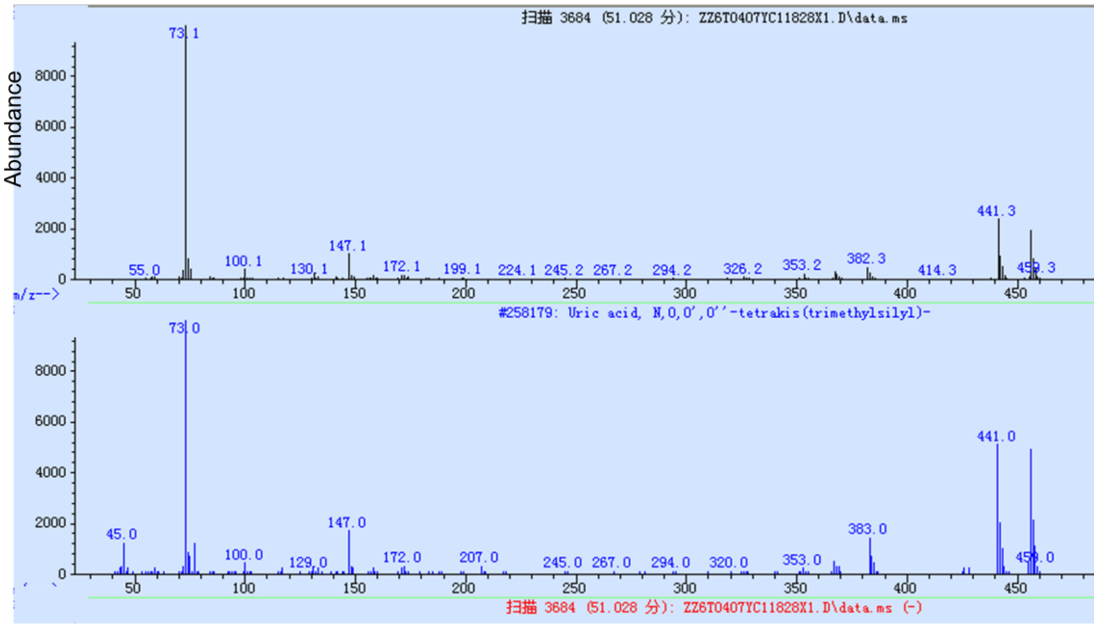

Fig 11 Electron-impact mass spectrum of uric acid TMS derivative.
